# Human Mitochondrial HMG-CoA Synthase Deficiency: Role of Enzyme Dimerization Surface and Characterization of Three New Patients

**DOI:** 10.3390/ijms19041010

**Published:** 2018-03-28

**Authors:** Beatriz Puisac, Iñigo Marcos-Alcalde, María Hernández-Marcos, Pilar Tobajas Morlana, Alina Levtova, Bernd C. Schwahn, Corinne DeLaet, Baiba Lace, Paulino Gómez-Puertas, Juan Pié

**Affiliations:** 1 Unit of Clinical Genetics and Functional Genomics, Department of Pharmacology-Physiology, School of Medicine, University of Zaragoza, CIBERER-GCV02 and ISS-Aragon, E-50009 Zaragoza, Spain; puisac@unizar.es (B.P.); mhmarcos@unizar.es (M.H.-M.); pilartobajas@yahoo.es (P.T.M.); 2 Molecular Modelling Group, Center of Molecular Biology “Severo Ochoa” (CSIC-UAM), Cantoblanco, E-28049 Madrid, Spain; imarcos@cbm.csic.es; 3 Faculty of Experimental Sciences, Francisco de Vitoria University, Pozuelo de Alarcón, 28223 Madrid, Spain; 4 Division of Medical Genetics, Department of Medicine, CHUM (Centre Hospitalier Universitaire de l’Université de Montréal) and Université de Montréal, Montreal, QC H2X 0A9, Canada; alevto@gmail.com; 5 Willink Metabolic Unit, Manchester Centre for Genomic Medicine, Saint Mary’s Hospital, Manchester University Hospitals NHS Foundation Trust, Manchester M13 9WL, UK; bernd.schwahn@mft.nhs.uk; 6 Hôpital Universitaire des Enfants Reine Fabiola, Avenue Crocq 15, B-1020 Brussels, Belgium; corinne.delaet@huderf.be; 7 Centre Hospitalier Universitaire de Québec, Québec City, QC G1V 4G2, Canada; baiba.lace@gmail.com

**Keywords:** mitochondrial HMG-CoA synthase, deficiency, mutations, enzyme dimerization, ketone bodies

## Abstract

Mitochondrial 3-hydroxy-3-methylglutaryl-CoA synthase deficiency (mitochondrial HMG-CoA synthase deficiency or mHS deficiency, OMIM #605911) is an inborn error of metabolism that affects ketone body synthesis. Acute episodes include vomiting, lethargy, hepatomegaly, hypoglycemia and dicarboxylic aciduria. The diagnosis is difficult due to the relatively unspecific clinical and biochemical presentation, and fewer than 30 patients have been described. This work describes three new patients with mHS deficiency and two missense mutations c.334C>T (p.R112W) and c.430G>T (p.V144L) previously not reported. We developed a new method to express and measure the activity of the enzyme and in this work the study is extended to ten new missense variants including those of our patients. Enzymatic assays showed that three of the mutant proteins retained some but seven completely lacked activity. The identification of a patient homozygous for a mutation that retains 70% of enzyme activity opens the door to a new interpretation of the disease by demonstrating that a modest impairment of enzyme function can actually produce symptoms. This is also the first study employing molecular dynamics modelling of the enzyme mutations. We show that the correct maintenance of the dimerization surface is crucial for retaining the structure of the active center and therefore the activity of the enzyme.

## 1. Introduction

Mitochondrial 3-hydroxy-3-methylglutaryl-CoA (HMG-CoA) synthase (mHS, EC 2.3.3.10) catalyzes the condensation reaction between acetyl-CoA and acetoacetyl-CoA in ketone body synthesis [1]. Ketone bodies are critical for providing energy to the brain during periods of fasting. Currently, there is a growing interest in the functioning of this enzyme, since it is also related to progression and apoptosis in some types of cancer [2,3].

mHS deficiency (OMIM #605911) is a rare autosomal recessive genetic disorder that is characterized by episodes of severe hypoketotic hypoglycemia, accompanied by vomiting, hepatomegaly, and lethargy, and which can progress to life-threatening coma [4]. A typical urine profile includes dicarboxylic aciduria and specific metabolites such as 4-hydroxy-6-methyl-2pyrone (4-HMP) that may only appear during periods of metabolic decompensation [5]. However, these findings overlap with those found in other inborn errors of metabolism, which makes the diagnosis difficult [6,7]. To date, mutations in the *HMGCS2* gene (GenBank NM_005518.2), which encodes mHS, have been described in fewer than 30 patients [5,8]. In order to confirm the pathogenicity of the mutations, we developed a method of expressing the enzyme and assaying its activity, which allowed the functional study of eight novel missense mutations found in patients with mHS deficiency [9].

Previous studies have already pointed out that mHS is a homodimeric enzyme [10], which was subsequently confirmed by the crystallization of the enzyme [11], with the two monomers oriented towards each other in an interface that encompasses 15% of the total molecular surface. Several patients with mHS deficiency have been described with mutations that were located on the described structure of the mHS [9,11], although the molecular mechanism for the lack of activity has not been studied in depth.

In this work, we describe the identification of three new patients of different ethnic origin with mHS deficiency. We provide the functional characterization of ten new missense variants including those of our patients, through the expression and measurement of enzymatic activity of the mutants. We also studied the molecular mechanism for the lack of enzyme activity in the mutants by performing computational simulations of their behavior with molecular dynamics procedures.

## 2. Results

### 2.1. Clinical and Biochemical Data in Patients

We report the clinical features of three patients with two new mutations in the *HMGCS2* gene that haven’t been reported before, and one described previously (Table 1).

#### 2.1.1. Patient 1

Male of Romanian origin with non-consanguineous parents. At 3 months of age and in the course of a febrile crisis accompanied by vomiting, hypoketosis and hypoglycemia occurred. During this period, lactate levels, acylcarnitine profile, amino acids and hormones were normal; however, the profile of organic acids in urine revealed the presence of dicarboxylic acids, while 3-hydroxybutyrate (3HB) and acetoacetate were at low levels. The β-oxidation test was performed on peripheral blood leukocytes giving a normal result.

#### 2.1.2. Patient 2

Male infant of South-Asian consanguineous ancestry admitted to hospital at the age of 11 months with an acute episode of vomiting, lethargy, hypoglycemia and relative hypoketonemia. His history was unremarkable apart from one unexplained episode of lethargy at the age of 3 months, when he had normal blood glucose. Free carnitines in blood were normal but for an increase in acylcarnitine. The profile of organic acids in urine showed high levels of medium chain dicarboxylic and hydroxydicarboxylic acids and only small amounts of ketones. Based on the presentation, mHS deficiency was suspected. The presence of 4-hydro-6-methyl-2pyrone in urine was confirmed after the genetic diagnosis.

#### 2.1.3. Patient 3

Male patient of French Canadian origin. The first symptoms appeared at the age of three years. He started to have convulsions and intensive sweating, and developed weakness and hypoglycemia without any provoking factors or infections. Plasma lactate and amino acids were normal; however, the profile of organic acids in urine revealed the presence of dicarboxylic acids. His development is age appropriate, and he is successfully treated by a diet consisting of frequent meals with high protein content.

### 2.2. Molecular Genetic Testing

In patient 1, the genetic analysis of the *HMGCS2* gene detected only the missense mutation c.334C>T in exon 2, which causes the protein change p.R112W. Inheritance was confirmed by analysis of the parents’ DNA. The MLPA (Multiplex Ligation Probe Analysis) search of large deletions or duplications gave a negative result.

In patient 2, next generation sequencing was performed covering a minimum depth of 50× of 100% of the target coding regions of transcripts of selected genes. This revealed an apparent homozygous point mutation, c.430G>T (p.V144L) in the *HMGCS2* gene. The mutation was confirmed by bidirectional Sanger sequencing, and both parents were found to be carriers for this same mutation.

In patient 3, 99.34% of coding exons were sequenced with coverage of at least 10× and the mutation c.1514G>A (p.R505Q) was identified in a homozygous state within the *HMGCS2* gene. It was confirmed by bidirectional Sanger sequencing and biparental inheritance was confirmed. This mutation has previously been described in a compound heterozygous state in a patient with mHS deficiency [5].

### 2.3. Protein Expression and Enzymatic Activity of mHS

The effect on enzyme function of the 10 missense mutations reported in patients was studied (8 recently reported: p.M146R, p.G169D, p.W185R, p.G232V, p.L266S, p.I407T, p.Y503C and p.R505Q; and two new mutations described in this work: p.R112W and p.V144L).

**Figure 1:**
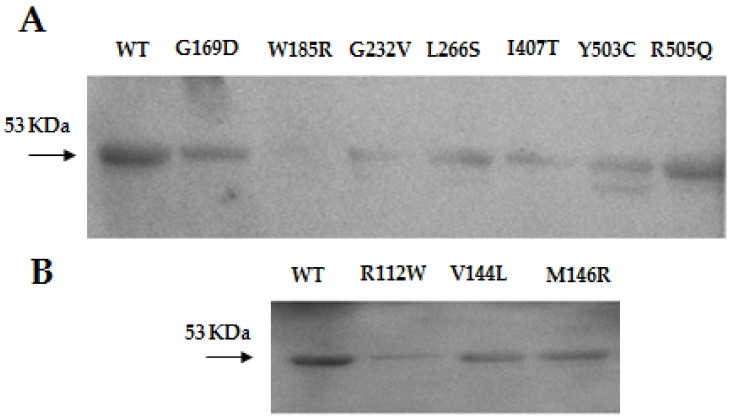
(**A**) Western blot of the human mHS protein (WT) and p.G169D, p.W185R, p.G232V, p.L266S, p.I407T, p.Y503C, p.R505Q previously reported and (**B**) p.R112W, p.V144L and p.M146R mutants.

**Figure 2:**
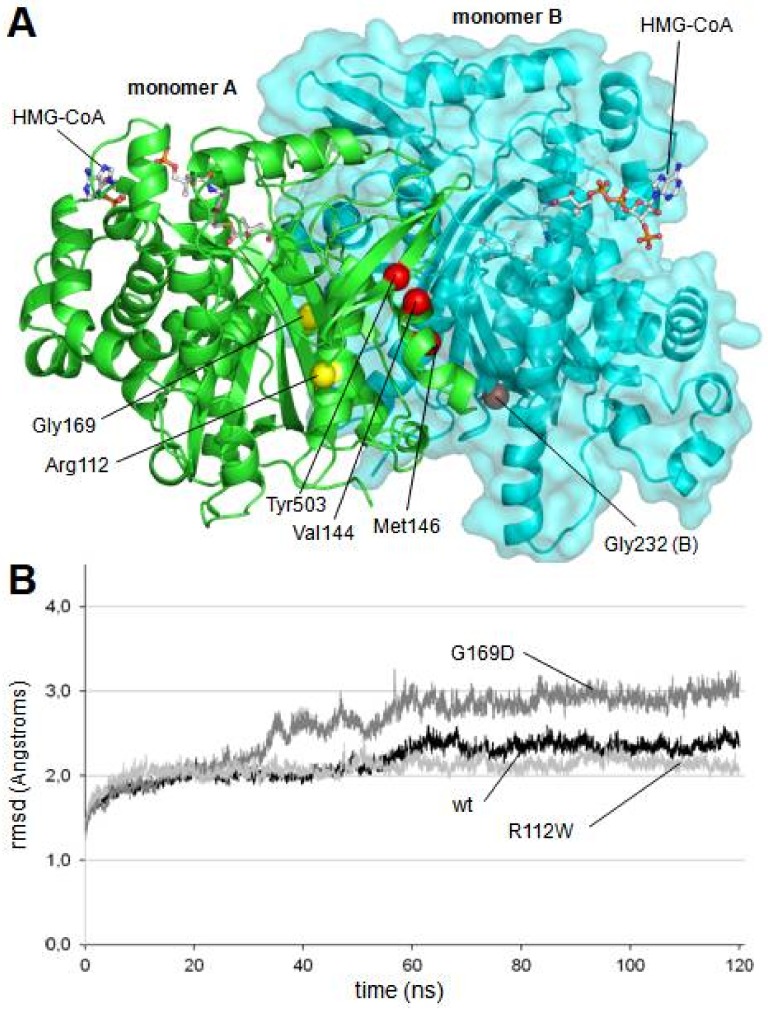
(**A**) Location of mutants in the dimer structure of human mitochondrial HMG-CoA synthase. Residues Val144, Met146, Gly232 and Tyr503, located in a cluster close to the dimerization surface, are colored in red. Mutated residues located away from the dimerization surface, Arg112 and Gly169, are colored in yellow. Location of two HMG-CoA molecules is indicated. (**B**) Root mean square deviation (rmsd) values measured over the unrestricted 120 ns of molecular dynamics trajectories of the wild type and the mutant mHS structures. For clarity purposes, only the trajectories defining the upper (p.G169D) and lower (p.R112W) limits, as well as the wild type trajectory, are depicted. Values of rmsd corresponding to all other trajectories (p.V144L, p.M146R, p.G232V and p.Y503C) are located between those limits.

The positions of the mutations in the protein structure are shown in Figure 2A. In vitro variants of the mHS protein were obtained by directed mutagenesis. After expression and purification of the mHS mutants, under the same conditions used for the wt mHS, all them were found to be expressed in soluble form. The mutated proteins exhibited moderate expression values relative to wild type (assigned 100%): p.R112W (22.4%), p.V144L (58.2%), p.M146R (59.3%), p.G169D (43.3%), p.G232V (18.0%), p.L266S (41.2%), p.I407T (26.6%), p.Y503C (34.7%) and p.R505Q (62.9%). Only p.W185R showed levels below 2% of the amount of protein comparing with the wild type (Figure 1A).

Only three mutants produced proteins exhibiting detectable enzymatic activity: active proteins: p.V144L (8.45 % compared with wild-type), p.Y503C (8.72%) and p.R505Q (70.6%) (Table 2).

### 2.4. Molecular Dynamics Simulations of mHS Mutants

To study the molecular reasons for the lack of enzymatic activity in the analyzed mHS mutants, some of them were selected to perform computational simulations of their behavior using molecular dynamics procedures. Those with negative presence in western blot analysis (Figure 1) were discarded, as they are supposed to produce unstable structures. Mutants p.L266S and p.I407T was not considered, as they are located in the core structure of the protein, very close to the active site, with a direct effect to catalytic residues. In addition, mutant p.R505Q was also discarded, as it was assumed that, with an in vitro enzymatic activity higher than 70% of the activity of wild type protein, no major differences were expected to be found in the dynamic analysis of its structure. Then, six different mHS mutants were selected to be analyzed using molecular dynamics: p.R112W, p.V144L, p.M146R, p.G169D, p.G232V and p.Y503C.

As indicated in Figure 2A, four of the mutated residues (Val144, Met146, Gly232 and Tyr503, red spheres in Figure 2A) are located forming a local cluster close to the interaction surface between the two subunits of the dimerized enzyme and far away from the active center of the enzyme.

To accomplish the simulation analysis, a structural model was obtained for each mutant using standard homology modeling procedures. The models of the six mutant proteins were subjected to 120 nanoseconds of free molecular dynamics simulation. As a control, the wild type dimer structure was also subjected to the same procedure. Stability of the alpha carbon trace during the entire simulation was continuously checked by measurement of root mean square deviation (rmsd) values. The rmsd values of all analyzed structures remained below 3.0 Å (Figure 2B) indicating the absence of dramatic changes in protein backbone, which is in consonance with the fact that all of them exhibited a positive result in western blot analysis.

**Figure 3:**
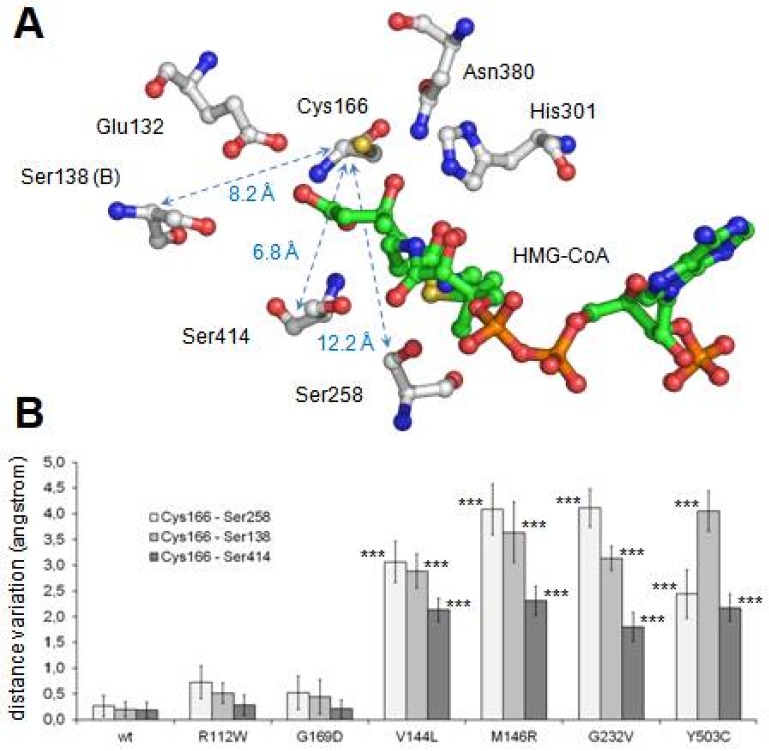

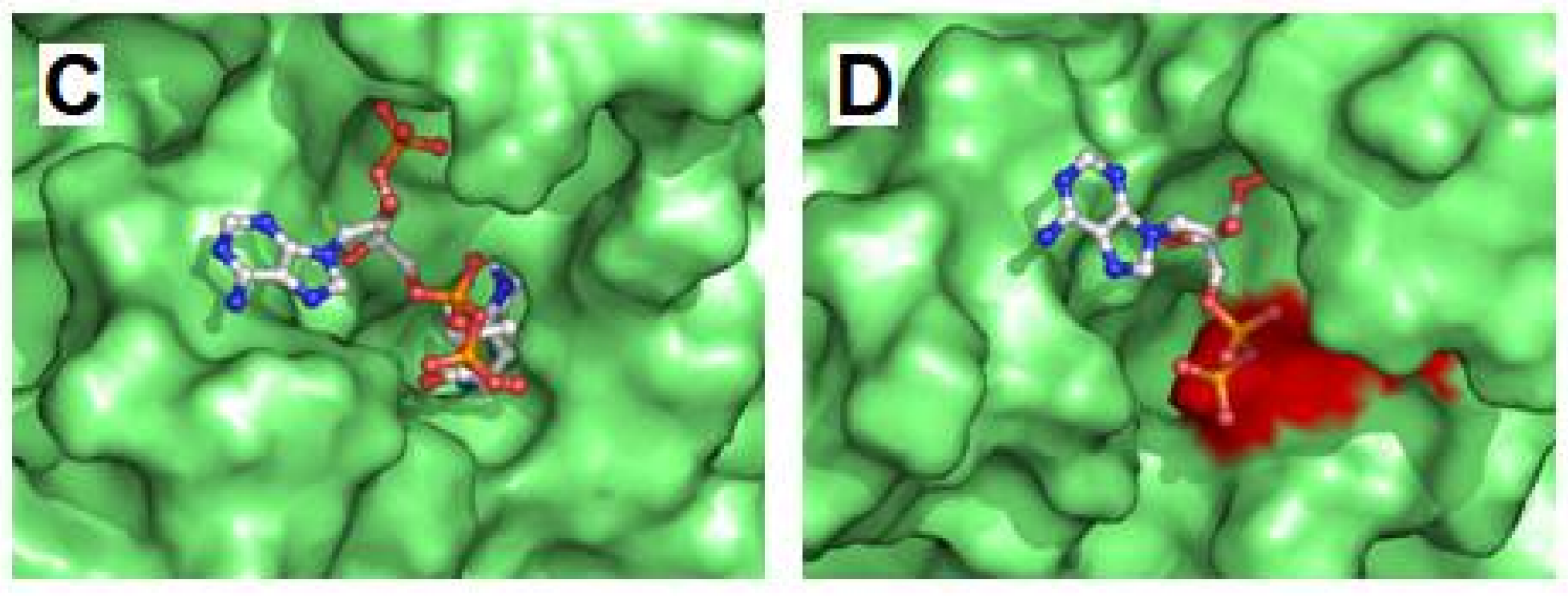
Analysis of maintenance of the structural integrity of the active center along molecular dynamics procedure. (**A**) Structure of mHS active center. The positions of residues implicated in the enzyme catalytic reactions (Glu132, Cys166, His301 and Asn380), as well as in the maintenance of the structure of the active site (Ser138, Ser258 and Ser414), are indicated. Note that residue Ser138 belongs to the counterpart subunit. Arrows identify distances indicative of the integrity of the active site structure. Distance values (in angstrom) corresponding to the mHS crystal structure (PDB id.: 2WYA) are shown. (**B**) Variation of the distances indicated in A for each mutant with respect to the average displacements in wild type structure. Values correspond to the mean ± standard deviation of the individual values measured every 20 ps during the last 20 ns of molecular dynamics. Student’s *t*-test was used to compare differences between each measurement and the one corresponding to the variation in the wild type structure (*** *p* < 0.001). (**C**,**D**) Substrate channel entrance of wild type mHS (**C**) and p.G169D mutant (**D**) after 120 ns of the free molecular dynamics procedure. The surface of residues blocking the tunnel access in p.G169D mutant is colored in red.

During the 120-ns-long simulation of all mutant structures, the maintenance of the correct shape of the active center cavity was monitored by the continuous measurement of the distances between the residues implicated in the enzymatic activity (Figure 3A). After comparison of all the obtained values (not shown), we found that the best descriptors of internal movements in the substrate cavity were the distances between the Cα of the catalytic residue Cys166 and the Cα of three serine residues located in the opposite wall of the substrate cavity (Figure 3A). In the case of the wild type protein, such distances remain constant along the entire molecular dynamics trajectory. To analyze the behavior of these three distances during the molecular dynamics trajectories of the mutants, individual values were collected every 20 ps along the last 20 ns of all trajectories (i.e., once the simulations have reached a stable stage). The difference between each value and the average value of the corresponding distance in the trajectory of the wild type protein was calculated. Mean and standard deviation of all measured differences are shown in Figure 3B. Student’s *t*-test was used to evaluate the statistical significance of the differences.

As shown in Figure 3B, changes in the relative distances of Cys166 to the three analyzed serine residues were not observed in two mutant proteins: p.R112W and p.G169D. In both cases, the mutated residue (Arg112 and Gly169, respectively) is located away from the dimerization surface (Figure 2A). Arginine 112 is a positively charged residue located in an alpha helix in the external surface of the protein while Glycine 169 is located in an alpha helix in the central core of the protein, close to the substrate tunnel. As shown in the Figure 3D, in the final stages of the molecular dynamics simulation of the p.G169D mutant, closing of the substrate channel entrance is observed, contrary to what occurs in the wild type protein (Figure 3C) and in the rest of the mutants analyzed (not shown).

## 3. Discussion

mHS deficiency is an ultra-rare disease that is difficult to diagnose, based on an unspecific clinical presentation and the absence of reliable biomarkers of the disease. In this work, three new patients with moderate ketosis and high levels of dicarboxylic acids in urine are presented. This can also be seen in genetic disorders or fatty acid oxidation and, sometimes, is secondary to liver failure [5,6]. It has been suggested that the presence of high levels of the metabolite 4-HMP could be a biomarker of the disease [5], but another report did not confirm this observation [13], and in our case, it was only present in one of the patients. Evaluation of 4-HMP is not always included in routine clinical analysis of organic acids in urine and can easily be overlooked. It is also possible that it can only be detected in acute episodes of hypoglycaemia and metabolic decompensation. Recently, a case has been described [8] with very low levels of high density lipoproteins (HDL) and high triglycerides, but plasma lipids in our patients have been normal.

In recent years, the implementation of massive sequencing techniques and especially of specific panels has greatly facilitated the diagnosis of the deficiency. This was also the case in patients 2 and 3, where the analysis of a panel of genes related to congenital disorders of metabolism was successfully employed to detect the novel mutation c.430G>T (p.V144L) and the already known p.R505Q [5] in a homozygous state. All these mutations seem to have an irregular distribution, with the variants of patient 1 and 2 located in exon 2, which is by far the largest, and that of patient 3 in exon 9. The distribution of mutations does not suggest the presence of a mutational hotspot.

Although genetic analysis has been an important step in the diagnosis of mHS deficiency, in most circumstances, confirmation requires the enzymatic assay of the mutated mHS. For this purpose, our group has developed a method to express and measure the mHS activity, which already allowed us to study eight known missense mutations [9]. In this work, the study is extended to ten new missense variants, including those of our patients. As expected, western blot detection of mutant proteins was variable, p.V144L, p.M146R, p.G169D, p.L266S, p.Y503C and p.R505Q showed moderate expression of the protein, whereas mutants p.R112W and pG232V exhibited low expression values, and the expression in mutant p.W185R was very low. These results agree with other studies that suggest that between 50% and 80% of the mutations responsible for monogenic diseases cause destabilization in protein folding with loss of solubility and early degradation [14,15]. In this case, between 20% and 50% of the missense mutations studied appeared to cause the same effect (Figure 1).

The results of the enzymatic assay showed that seven mutants lack activity, while three of them demonstrate positive results: two mutants with reduced activity, approx. 8% of wt. (p.V144L, 0.10 ± 0.02 U/mg protein, p.Y503C, 0.11 ± 0.02 U/mg protein), and a third one with very high activity (p.R505Q; 0.86 ± 0.06 U/mg of protein) in the order of 70% of wt. This is the first time that a mutant with more than 30% activity [9] has been found. In the structure of the protein, Arg505 residue is found on the outer surface of the protein, away from the dimerization surface and the active sites. It interacts with Glu115 residue also on the protein surface. The mutation of Arg505 to Gln, another polar residue, although not charged, is supposed to maintain the interaction with Glu115 through the amino group of Gln. The effect of this mutation can then be expected to be mild, corresponding to the 70.6% specific activity observed in comparison with the wild protein.

The lack of enzymatic activity was studied by molecular dynamics simulation. Results indicate that four of the analyzed mutants (p.V144L, p.M146R, p.G232V and p.Y503C), all of them located in a cluster near the dimerization surface and away from the active center (Figure 1A), exhibit a similar and significant influence on the geometry of the active center (Figure 3B). The observed variation is large enough (in some cases with differences of more than 4.5 angstroms with respect to the wild type values) to cause the disorganization of the active center and, consequently, the inactivation of the enzyme activity. The shared spatial position of residues Val144, Met146, Gly232 and Tyr503 in the dimerization surface and their common influence on the arrangement of the active site strongly suggest that this surface patch plays an important role in the correct performance of the protein. Changes in this site would result in the complete lack of enzyme activity.

The mutated residues Arg112 and Gly169 are located away from the dimerization surface, and a different mechanism must apply here. Mutation of arginine 112 to a hydrophobic tryptophan was initially predicted to seriously affect protein structure. Surprisingly, its behavior during molecular dynamics trajectory indicated that the alpha carbon trace was maintained more stable than the trace of the wild type enzyme (Figure 2B), and no major changes were observed in the substrate channel or in the residues of the active center. Molecular dynamics analysis was, on this occasion, inconclusive. Glycine 169 is located in a close position (less than 5.0 angstroms) to the catalytic residue Cys166 and thus influences the residues in the substrate channel. In the G169D mutant, the substitution of Gly169, a small amino acid, by aspartic acid, a large and charged residue, generated a set of critical changes in the structure of the substrate cavity, leading to the closure of the channel entrance at the end of the molecular dynamics simulation (Figure 3C,D).

In summary, the results obtained using both in vitro analysis and computational simulations of mHS mutants located far from the substrate cavity, indicate that the maintenance of the dimerization surface is also crucial for keeping the structure of the active center. It is interesting to note that in this case, the effect of the location of the mutated residues (in rigid central versus flexible external regions [16]) on enzyme activity is variable. In addition to the amino acids located on the dimerization surface, some residues located on the external surface cause a complete lack of activity (p.R112W), while others do not (p.R505Q). This difference is not observed in mutants in the protein core: all of them promote a clear reduction of enzyme activity (mutants p.G169D, p.L266S and p.I407T). With regard to the genotype-phenotype relationship, the deficiency of mHS was compared to that of mitochondrial HMG-CoA lyase (HL), an enzyme that follows it in the synthesis pathway of ketone bodies [17]. Both deficiencies share features of their clinical presentation although in the case of HL it is usually more serious [18]. The enzymatic assay of HL mutants invariably demonstrates a complete or almost complete lack of enzyme activity, which suggests that the manifestation of the disease is more dependent on precipitating circumstances than to the type of mutation [17]. However, the first activity study of the mutants of the mHS enzyme revealed that one of them had 29% activity [9].

Since mHS is a dimeric enzyme, the ideal model for establishing genotype-phenotype relationships is homozygous patients. In this work, two homozygous patients are presented with a mutation that produces partial failure of the enzyme. In patient 2, the enzyme retains 8.45% activity. The patient, however, had a moderately severe but classical clinical presentation with a suggestive profile of acylcarnitines in plasma and organic acids in urine including even as the presence of the 4-HMP biomarker. The symptoms of vomiting, lethargy and a discrete encephalopathy cannot be considered as mild. We were already surprised in a previous study to find a symptomatic patient with 29% of residual mHS activity [9]. Here, we present a symptomatic patient homozygous for the R505Q mutation, which preserves 70% of mHS activity. The clinical symptoms of this patient, although moderate, included hypoglycaemia, abnormal acylcarnitines in plasma and dicarboxylic acids in urine. He presented, however, with adequate ketosis. This finding is relevant and suggests that slight decreases in the activity of the mHS enzyme accompanied by metabolic stress situations and low blood glucose levels may trigger a hypoglycaemic manifestation. On the other hand, it is possible that this patient has idiopathic ketotic hypoglycaemia due to accelerated starvation, and the genetic variation identified is an incidental finding due to an ascertainment bias. However, it must be considered that this is the second patient described with this mutation and mild clinical manifestations of mHS deficiency, and the effect of the mutation has been confirmed with the in vitro assay of enzymatic activity. The role of the R505Q mutation in symptomatology is unclear. The formation of unpaired heterodimers cannot be ruled out when the mutation is found in a compound heterozygous state [5]. In addition, other unknown functions of this residue, e.g., the transport of the protein to the mitochondria, may provide a possible explanation for the symptoms.

In conclusion, this paper describes three new patients with mHS deficiency and the first study by molecular dynamics of mHS enzyme mutations. We identified the crucial importance of the correct maintenance of the dimerization surface for keeping the structure of the active center and, therefore, the activity of the enzyme. The finding of a homozygous patient for a mutation that retains 70% of the enzyme’s activity opens the door to a new interpretation of the disease in which a small involvement of the enzyme can produce the deficiency.

## 4. Materials and Methods

### 4.1. Patients

This study includes three new patients with mitochondrial HMG-CoA synthase deficiency. Ethical recommendations of the Declaration of Helsinki were followed. Patients’ parents or guardians signed the informed consent to participate in the study.

### 4.2. Genetic Diagnosis 

In patient 1, genomic DNA was extracted from a blood sample of patients with standardized protocols. Coding regions and flanking intronic sequences of *HMGCS2* (ten exons) were screened for pathological variants by PCR amplification of blood genomic DNA [9]. The PCR products were purified with USB ExoSAP-IT PCR Product Cleanup (Affymetrix, Santa Clara, CA, USA) following the manufacturer’s instructions and sequenced in a ADN3130 Genetic Analyzer (Applied Biosystems, Foster City, CA, USA). Parental genotypes were screened to assess whether the variant was de novo or inherited.

In patient 2, Targeted Next Generation Sequencing was applied. Enrichment of Genomic DNA was performed with a Sure Select custom target enrichment kit (Agilent, Santa Clara, CA, USA) for the HiSeq 2500 (lllumina, San Diego, CA, USA) system, following the manufacturer’s protocols. The target enrichment design consists of the coding region of transcripts, including the immediate splice sites (+1–5 bases), for 227 genes associated with metabolic disorders. The samples were sequenced using a HiSeq 2500 (lllumina), in accordance with the manufacturer’s protocols. For the selected genes, sequence data was mapped with GenomeAnalysisToolKitLite-v2.0.39 (Broad Institute, Cambridge, CA, USA), using hgl9 human genome as a reference. Known polymorphisms were subsequently filtered out of the data obtained using bioinformatic analysis.

In patient 3, Genomic DNA was isolated and enriched for the coding exons of targeted genes using hybrid capture technology. Prepared DNA libraries were then sequenced using a Next Generation Sequencing technology. 99.34% of coding exons had been sequenced, with coverage at least 10×.

### 4.3. mHS Protein Expression in E. coli

cDNA encoding mHS without the signal peptide was amplified from liver by PCR and cloned into the expression plasmid pMAL-c2x as described in [9]. Mutations were introduced on pMAL-c2x-mHS using the QuickChange™ Site-Directed Mutagenesis Kit (Agilent) according to the manufacturer’s instructions. *E. coli* strain BL21 expressing MBP-mHS wt and MBP-mHS of different mutants was grown in LB medium to an A600 of 0.8–1.0 at 37 °C. Optimal protein expression was induced with 0.3 mM IPTG at 20 °C for 18 h. Cells were recovered, lysed and disrupted by thermal shock. The soluble fraction containing the MBP-mHS fusion proteins were loaded into an amylase affinity column, washed, and finally eluted from the affinity resin using a buffer containing the protease factor Xa [9].

### 4.4. Western Blot Analysis

Purified proteins were quantified by Bradford’s method. 5 µg of each protein sample were subjected to SDS-PAGE electrophoresis, and transferred to a 0.45 PVDF membrane. Membranes were incubated with a 1:500 dilution of a monoclonal antibody (MO6) against mHS 424–508 aminoacids (Abnova, Taipei City, Taiwan) and a 1:1000 dilution of a secondary anti-mouse antibody. The blots were developed with the Immobilon Western Chemiluminescent HRP Substrate (Millipore) kit. The images obtained were processed using Adobe Photoshop 5.0. The relative amounts of mutated mHS protein compared with the wild type (assigned 100%) were determined using the software Image Studio Lite Analysis Software for Western Blots.

### 4.5. Enzymatic Activity

Mitochondrial HMG-CoA synthase activity was determined measuring the amount of acetoacetyl-CoA by spectrophotometry at 304 nm, as previously described in the literature [9]. Each experiment was performed in triplicate.

### 4.6. Structure Modeling of mHS Mutants 

3D structures of mHS mutants were modeled using as template the structure of wild type human protein crystalized in presence of HMG-CoA (Protein Data Bank ID: 2WYA) [11]. Models were built using the SWISS-MODEL server (http://swissmodel.expasy.org), and their structural quality was within the range of those accepted for homology-based structure (Anolea/Gromos/QMEAN4) [19]. Prior to molecular dynamics procedures, models were energy minimized using the GROMOS 43B1 force field implemented in DeepView (http://spdbv.vital-it.ch/), using 500 steps of steepest descent minimization followed by 500 steps of conjugate-gradient minimization.

### 4.7. Molecular Dynamics Simulations

Modeled structures were subjected to molecular dynamics simulation using the AMBER14 molecular dynamics package [20]. The 3D structures were solvated with periodic cuboid pre-equilibrated solvent boxes using the LEaP module of AMBER, with 12 Å as the shortest distance between any atom in the protein and the periodic box boundaries. Free MD simulations were performed using the PMEMD program of AMBER and the parm99 force field [20], using the SHAKE algorithm. Systems were initially relaxed over 15,000 steps of energy minimization with a cut-off of 12 Å. Simulations were then started with a 20 ps heating phase, raising the temperature from 0 to 300 K in 10 steps. During minimization and heating, the Cα trace dihedrals were restrained and gradually released in an equilibration phase in which the force constant was gradually reduced to 0 over 200 ps. After the equilibration phase, 120 ns of free molecular dynamics simulations were obtained for wild type and mutant structures. Root mean square deviation (rmsd) of Cα trace was monitored along the trajectories, measuring it every 20 ps. In addition, a set of distances between the Cα of residues in the active center was also continuously measured to analyze the adequate spatial arrangement for enzymatic reaction. Molecular dynamics trajectories were analyzed using VMD software [21]. Figures were generated using the Pymol Molecular Graphics System (Schrödinger, LLC, Portland, OR, USA).

## Figures and Tables

**Table 1:** Summary of clinical and biochemical findings of the patients.

Features		Patient 1	Patient 2	Patient 3
**Clinical Manifestations**	Age at Presentation	3 months	11.5 months	3 years
	Vomiting	+	+	−
	Coma	−	−	−
	Lethargy	−	+	−
	Abnormal Breathing	−	−	−
	Hepatomegaly	−	−	−
	Developmental Delay	−	−	−
	Encephalopathy	−	+ (mild)	−
**Biochemical Data (plasma or serum)**	Hypoglycemia	+	+	+
	Metabolic Acidosis	−	−	−
	Hypoketonemia	+	+	−
	Elevated Lactate	−	−	−
	Elevated Free Carnitine	−	−	−
	Abnormal Acylcarnitines	−	+	+
	Elevated Ammonia	−	−	−
	Raised Transaminases	−	+	+
**Biochemical Data (urine)**	Ketonuria	+ (low)	+ (low)	+ (low)
	Dicarboxylic Aciduria	+	+	+
	Hydroxydicarboxylic Aciduria	?	+	?
	* 4-HMP	?	+	?

+ present, − absent, ? unknown, * 4-hydroxy-6-methyl-2-pyrone (biomarker).

**Table 2:** Functional study of the reported missense mutations in the *HMGCS2* gene from patients with mHS deficiency.

Mutation	Exon	Protein Effect	Specific Activity (U/mg)	Ref.
-	-	Wild type	1.24 ± 0.01 (100%)	-
c.334C>T	E2	p.R112W	n.d.	This work
c.430G>T	E2	p.V144L	0.1 ± 0.01 (8.45%)	This work
c.437T>G	E2	p.M146R	n.d.	Levtova et al. 2018 [12]
c.506G>A	E2	p.G169D	n.d.	Pitt et al. 2015 [5]
c.553C>T	E4	p.W185R	n.d.	Pitt et al. 2015 [5]
c.695G>T	E4	p.G232V	n.d.	Pitt et al. 2015 [5]
c.797T>C	E4	p.L266S	n.d.	Pitt et al. 2015 [5]
c.1220T>C	E7	p.I407T	n.d.	Pitt et al. 2015 [5]
c.1508A>G	E9	p.Y503C	0.11 ± 0.02 (8.72%)	Pitt et al. 2015 [5]
c.1514G>A	E9	p.R505Q	0.86 ± 0.05 (70.6%)	Pitt et al. 2015 [5]

n.d.: not detectable.
